# Current Status, Challenges, and Future Directions in Cardiac Rehabilitation

**DOI:** 10.3390/medicina60030388

**Published:** 2024-02-25

**Authors:** Francisco Epelde

**Affiliations:** Department of Internal Medicine, Hospital de Sabadell, Universitat Autónoma of Barcelona, 08208 Barcelona, Spain; fepelde@gmail.com

Cardiovascular diseases (CVDs) continue to pose a significant global health challenge, representing a leading cause of morbidity and mortality worldwide. The epidemiological landscape of CVDs demands urgent attention, as their prevalence and impact persistently burden societies across the globe [1]. The World Health Organization (WHO) estimates that CVDs are responsible for nearly 18 million deaths annually, accounting for approximately 31% of all global deaths. This staggering figure underscores the pervasive nature of these diseases, affecting individuals from diverse demographics and socioeconomic backgrounds. The burden of CVDs is not limited to high-income countries; low- and middle-income nations are experiencing a disproportionate rise in CVD-related morbidity and mortality [2].

The burden of disability arising from cardiovascular diseases has reached alarming proportions, with a substantial number of individuals globally experiencing limitations in daily activities due to heart-related conditions. The World Health Organization (WHO) estimates that CVDs are a leading cause of disability-adjusted life years (DALYs), emphasizing the profound impact on individuals' functional abilities and overall quality of life [3]. Cardiac events, such as heart attacks and heart failure, can lead to a range of physical impairments that significantly affect mobility and daily functioning. Chronic conditions like heart failure may result in fatigue, shortness of breath, and decreased exercise tolerance, limiting patients' ability to engage in routine activities. This functional impairment not only affects the individuals directly but also places a considerable burden on caregivers and healthcare systems.

Beyond the physical limitations, cardiovascular-related disabilities also exert a notable psychosocial toll on affected individuals. Depression, anxiety, and a diminished sense of well-being are common among those grappling with the challenges of cardiovascular disabilities. These psychosocial factors can further exacerbate the overall burden of disease, hindering rehabilitation efforts and impeding the return to a fulfilling life post-cardiac event. Acknowledging the impact of cardiovascular-related disability underscores the importance of comprehensive rehabilitation services. Multidisciplinary approaches, including physical therapy, cardiac rehabilitation programs, and psychosocial support, are crucial components in addressing the diverse needs of individuals disabled by cardiopathies. Access to these services should be prioritized globally to ensure equitable care and support for affected populations.

Efforts to address the disability burden imposed by cardiovascular diseases should align with global initiatives promoting cardiovascular health. Collaborative endeavors such as the American Heart Association's patient education programs and the European Society of Cardiology's initiatives on cardiac rehabilitation play a pivotal role in enhancing awareness, prevention, and rehabilitation for those with cardiovascular-related disabilities [4,5].

Cardiac rehabilitation (CR) has emerged as a pivotal component in the continuum of care for individuals with cardiovascular diseases, offering a holistic approach to recovery and prevention. However, despite its proven benefits, the field faces a myriad of challenges that impede its widespread effectiveness. This editorial seeks to shed light on the key challenges facing cardiac rehabilitation today and emphasizes the need for collaborative efforts to address these issues. Despite the wealth of evidence supporting the positive impact of cardiac rehabilitation, there exists a significant gap between the number of eligible patients and those actually participating in CR programs. Numerous studies highlight the underutilization of CR, with factors such as lack of awareness, geographical barriers, and physician referrals contributing to low participation rates [6,7].

Access to cardiac rehabilitation is often hindered by socioeconomic factors, creating disparities in participation among different demographic groups. Financial constraints, transportation issues, and the limited availability of rehabilitation programs in certain areas disproportionately affect vulnerable populations, exacerbating existing health inequities. A one-size-fits-all approach may not effectively address the diverse needs of individuals undergoing cardiac rehabilitation. Tailoring interventions to the unique characteristics and preferences of patients is crucial for maximizing engagement and long-term adherence to lifestyle modifications. The psychological impact of cardiovascular diseases is often underestimated. Cardiac rehabilitation programs must integrate mental health support to address anxiety, depression, and the emotional challenges associated with cardiac events. Neglecting mental health aspects may hinder overall recovery and rehabilitation outcomes. The rapid evolution of technology offers opportunities for innovation in cardiac rehabilitation, but it also presents challenges. Access to technology, digital literacy, and concerns about data security may hinder the widespread adoption of tele-rehabilitation and other tech-driven solutions [8]. The integration of technology into cardiac rehabilitation holds promise for overcoming some of the existing challenges. Tele-rehabilitation, mobile applications, and wearable devices provide opportunities for remote monitoring, personalized interventions, and enhanced patient engagement. Healthcare systems should embrace these technological advancements to extend the reach of cardiac rehabilitation programs and tailor interventions to individual needs [9].

Cardiac rehabilitation should not be limited to physical exercise but should encompass a multidisciplinary approach addressing the diverse needs of patients. Nutritional counseling, mental health support, and comprehensive lifestyle interventions should be integral components of cardiac rehabilitation programs to address the holistic well-being of individuals with cardiovascular diseases. While cardiac rehabilitation has made significant strides in improving the lives of individuals with cardiovascular diseases, challenges persist in ensuring widespread access and adoption. A concerted effort is required from healthcare providers, policymakers, and the community to bridge the existing gaps and optimize the delivery of cardiac rehabilitation services. Embracing technology, addressing socioeconomic disparities, and adopting a holistic patient-centered approach will contribute to the evolution of cardiac rehabilitation, ensuring its continued impact on cardiovascular health in the years to come.

The future of cardiac rehabilitation lies in the customization of interventions to meet the unique needs of each patient. With the advent of genomics and personalized medicine, tailoring CR programs based on individual genetic, lifestyle, and clinical factors will become increasingly feasible, maximizing the effectiveness of interventions [10]. Artificial Intelligence holds immense potential in revolutionizing cardiac rehabilitation. AI algorithms can analyze vast datasets, predict patient outcomes, and provide real-time adaptive feedback. From personalized exercise prescriptions to continuous monitoring, AI-driven solutions could significantly enhance the efficacy and accessibility of CR programs [11]. Virtual and augmented reality technologies offer immersive experiences that can be harnessed to enhance exercise regimens and therapeutic interventions in CR. These technologies have the potential to increase patient engagement, motivation, and adherence to rehabilitation programs [12]. The COVID-19 pandemic has accelerated the adoption of telehealth, and cardiac rehabilitation is no exception. Tele-rehabilitation allows for the remote monitoring of patients, virtual consultations, and the delivery of rehabilitation programs in the comfort of patients' homes, overcoming geographical barriers and improving accessibility [13]. Behavioral economics principles can be leveraged to design interventions that optimize patient motivation and adherence. By understanding and addressing the psychological and behavioral aspects of lifestyle changes, cardiac rehabilitation programs can be better tailored to promote sustained behavior modification [14].

The future of cardiac rehabilitation is dynamic and holds the promise of transforming the way we approach cardiovascular health. By embracing personalized and precision medicine, integrating artificial intelligence, harnessing virtual and augmented reality, expanding tele-rehabilitation, and incorporating insights from behavioral economics, we can create a new era in cardiac rehabilitation. The collaboration between healthcare professionals, researchers, and technology innovators will be paramount in ensuring that these advancements translate into tangible improvements in patient outcomes and contribute to a healthier cardiovascular landscape. The journey ahead is one of innovation, inclusivity, and personalized care, charting a course towards a brighter future for cardiac rehabilitation.

## References

[1] World Health Organization (2022). Cardiovascular Diseases (CVDs). https://www.who.int/news-room/fact-sheets/detail/cardiovascular-diseases-(cvds).

[2] Joseph P., Leong D., McKee M., Anand S.S., Schwalm J.D., Teo K., Mente A., Yusuf S. (2017). Reducing the Global Burden of Cardiovascular Disease, Part 1: The Epidemiology and Risk Factors. Circ. Res..

[3] Ralapanawa U., Sivakanesan R. (2021). Epidemiology and the Magnitude of Coronary Artery Disease and Acute Coronary Syndrome: A Narrative Review. J. Epidemiol. Glob. Health.

[4] American Heart Association Patient Education. https://www.heart.org/en/health-topics/consumer-healthcare/faq/patient-education.

[5] European Society of Cardiology Cardiac Rehabilitation. https://www.escardio.org/Education/Practice-Tools/Cardiac-rehabilitation-resources.

[6] Anderson L., Oldridge N., Thompson D.R., Zwisler A.D., Rees K., Martin N., Taylor R.S. (2016). Exercise-based cardiac rehabilitation for coronary heart disease: Cochrane systematic review and meta-analysis. J. Am. Coll. Cardiol..

[7] Baman J.R., Sekhon S., Maganti K. (2021). Cardiac Rehabilitation. JAMA.

[8] Taylor R.S., Dalal H.M., McDonagh S.T.J. (2022). The role of cardiac rehabilitation in improving cardiovascular outcomes. Nat. Rev. Cardiol..

[9] Wongvibulsin S., Habeos E.E., Huynh P.P., Xun H., Shan R., Porosnicu Rodriguez K.A., Wang J., Gandapur Y.K., Osuji N., Shah L.M. (2021). Digital Health Interventions for Cardiac Rehabilitation: Systematic Literature Review. J. Med. Internet Res..

[10] Tada H., Fujino N., Nomura A., Nakanishi C., Hayashi K., Takamura M., Kawashiri M.A. (2021). Personalized medicine for cardiovascular diseases. J. Hum. Genet..

[11] Sotirakos S., Fouda B., Mohamed Razif N.A., Cribben N., Mulhall C., O’Byrne A., Moran B., Connolly R. (2022). Harnessing artificial intelligence in cardiac rehabilitation, a systematic review. Future Cardiol..

[12] Jung C., Wolff G., Wernly B., Bruno R.R., Franz M., Schulze P.C., Silva J.N.A., Silva J.R., Bhatt D.L., Kelm M. (2022). Virtual and Augmented Reality in Cardiovascular Care: State-of-the-Art and Future Perspectives. JACC Cardiovasc. Imaging.

[13] Ramachandran H.J., Jiang Y., Tam W.W.S., Yeo T.J., Wang W. (2022). Effectiveness of home-based cardiac telerehabilitation as an alternative to Phase 2 cardiac rehabilitation of coronary heart disease: A systematic review and meta-analysis. Eur. J. Prev. Cardiol..

[14] Tian Y., Deng P., Li B., Wang J., Li J., Huang Y., Zheng Y. (2019). Treatment models of cardiac rehabilitation in patients with coronary heart disease and related factors affecting patient compliance. Rev. Cardiovasc. Med..

